# Intersections Around Ambivalent Sexism: Internalized Homonegativity, Resistance to Heteronormativity and Other Correlates

**DOI:** 10.3389/fpsyg.2020.608793

**Published:** 2020-12-03

**Authors:** Miguel Ángel López-Sáez, Dau García-Dauder, Ignacio Montero

**Affiliations:** ^1^Department of Psychology, Social Psychology Area, Rey Juan Carlos University, Alcorcón, Spain; ^2^Department of Social Psychology and Methodology, Faculty of Psychology, Autonomous University of Madrid, Madrid, Spain

**Keywords:** ambivalent sexism, internalized homonegativity, heteronormative resistances, political conservatism, *ex post* facto study

## Abstract

This article explores the connections between the construct of sexism and other sociodemographic and attitudinal variables, such as internalized homonegativity and heteronormative resistances, among psychology students. Both unrefined and inferential analyses were used with a representative sample of 841 psychology students from public universities in Madrid. Results showed higher levels of sexism, internalized homonegativity and low resistances to heteronormativity among groups of men, heterosexuals and conservatives. Interactions were found that showed a higher degree of hostile sexism in: heterosexual people with respect to LGB and heterosexual men with respect to heterosexual women. Also, interactions were found to show a greater degree of heteronormative resistance in: LGB people with respect to heterosexuals and left-wing women with respect to right-wing women. Correlations with sexism varied according to gender identity and sexual orientation. In addition, heteronormative resistances correlated negatively with sexism, while some components of internalized homonegativity correlated positively. Political affiliation was the most frequent predictor of sexism. The results highlight the need for an intersectional approach to understanding the phenomenon of sexism.

## Introduction

Almost 25 years after the Association of American Colleges and Universities showed that sexual assault on women on college campuses was related to a hidden decrease in gender equality (ACU, 1978), current data reaffirm that inequality remains an unresolved issue. In 2018, 236 allegations and reports of sexual abuse were presented in Spanish universities (Jara, 2018). However, various studies have indicated that many more female students have experienced abuse, but without reporting it (Blahopoulou et al., 2012; Igareda and Bodelón, 2014; Onetti et al., 2018). For that reason, it is important to examine the beliefs and discourses that articulate this abusive behavior against women and are key to its normalization (Blahopoulou et al., 2012). Moreover, such a study is required by current law, and a commitment has been made to education and research initiatives at the international level (CEDAW, 2015), the European level (Programa, 2020; EU, 2013) and in Spain (Law 14/11; BOE, 2011). All of this is particularly important for certain groups like future psychology graduates in terms of their eventual professional work, as laid out in the Psychology White Paper (ANECA, 2005).

Another sector of the population that has been subject to sexual abuse on campus are lesbians (L), gays (G), bisexuals (B), and trans (T), as shown by national and international studies (Hughes, 2018; Rebollo et al., 2018; Biglia and Cagliero, 2019). The visibility of this abuse is low, as it is estimated that only between 16 and 40% of these cases are reported (Ahmed, 2018; Biglia and Cagliero, 2019). Their incidence is minimalized in academic institutions, which scarcely recognize them and keep no detailed record of their prevalence (Ahmed, 2018; Biglia and Cagliero, 2019). The motivating factor for the abuse is a sexual orientation, identity or gender expression contrary to the established normativity (cisgender and heterosexual), and not only among LGBT individuals. This forces the person to engage in so-called “passing” to remove any possible suspicion of sexual or gender dissidence (Bachmann and Gooch, 2018; Garvey et al., 2018).

This type of abuse is related to sexism, since LGBTphobia is based on monitoring that which diverges from the spheres of traditional masculinity and femininity. For example, many LGBT people are insulted or scorned because their features or behaviors are considered improper, extending the sexism reaction to men and women who deviate from traditional norms in some aspect of their identity, gender expression and/or sexuality. In Spanish, the genesis of the term “*marica*” – originally a word formed from the diminutive of the name María and meaning “effeminate,” but now a common derogatory term for a homosexual – lays the groundwork for an initial explanation of the connections between sexism and homonegativity (Díaz, 2014). In fact, the discomfort that heterosexual men feel toward homosexual men is, really, a lack of comfort with the expression of female gender (Parrott et al., 2002). Likewise, the literature confirms an alliance between sexist and LGBT-phobic attitudes (López-Sáez et al., 2020) and the resulting violence (Biglia and Cagliero, 2019). Sexism allows for a hierarchization of privilege, where heterosexual, cisgender men are at the top and everybody else is beneath them (Connell, 2014; Glick et al., 2015).

The existence of negative attitudes toward LGBT people is mediated by many factors, including gender identity, knowledge of and contact with the community, political ideology, age, religiousness and sexism, and it is sexism that appears to be one of the most determinant predictors of the degree of LGBTphobia and, particularly, homophobia (Davies, 2004; Warriner et al., 2013; Scandurra et al., 2017). Furthermore, sexism as a prejudice has a shared evolution with homophobia, becoming increasingly subtle and sophisticated (Quiles et al., 2003). Sexism seems to influence not only perceptions against non-heterosexuals, but also a negative self-concept among heterodissident individuals themselves. Therefore, sexism helps to guarantee the prevalence of the traditional spheres of femininity and masculinity through the interiorization of the rejection of the non-heterosexual orientation itself. Through this internalization, the rupture that men and women make with respect to normative expressions, roles and desires is punished.

Weinberg (1972) coined the term “homophobia based on self-loathing” to refer to these internalized negative beliefs. According to Herek et al. (2015), the term has been evolving and there are several nomenclatures that are used synonymously, such as internalized heterosexuality, internalized homophobia and internalized homonegativity (the latter two are used interchangeably in the article). The use of one term or another corresponds to discussions about nuances that have been resolved by introducing a meaning to the signifier that includes a range of connative inferences, cognitions and negative emotions toward the homosexual (López-Sáez et al., 2020). In a heterosexist society, where sexual stigma against heterodissidents is part of the social structure of values (Herek, 2007, 2009), it is not surprising that this structure is internalized as its own through a socialization where the expectations of being heterosexual prevail (Herek et al., 2015). As observed by Allport (1954), a negative consideration of others has adverse effects that affect one’s character. When this occurs, internalized stigma is directed toward self-concept and the experience of desire, generating an associated discomfort for the person (Crocker et al., 1998). Such internal discomfort would be suffered as part of a stressful process resulting from belonging to a minority. Having a dissident sexual orientation can be linked to distal or proximal stress processes, where the former refers to external stressors, such as heterosexist and heteronormative social beliefs, and the latter depends on the self and is related to the assumption of those beliefs (Meyer, 1995, 2003). In the development of identities that challenge gender binarity in some of its terms, proximal stressors generate continued feelings of invalidity and unacceptance (López-Gómez and Platero, 2018; Scandurra et al., 2019a). Therefore, self-stigma and proximal stressors have aversive consequences on physical and psychological health at the intra- and interpersonal levels (Bradford et al., 2013; McLaren, 2016; Scandurra et al., 2017; Moody et al., 2018), as much or more than distal stressors and external stigma (Pachanakis and Bränström, 2018). Because of this, LGB people may hide their orientation or identity (by passing) to cushion the negative distal effects. This adaptation seeks to meet the expectations of the regulatory spheres of gender and sexuality (Scandurra et al., 2019b). However, such actions only generate a kind of persecutory hysteria (Anderson, 2009; Vitelli, 2015) by reinforcing self-stigma and proximal stressors through self-monitoring (Meyer, 2007; Beemyn and Rankin, 2011). For example, the culture of passing is common in spaces like universities, where students adapt their behavior to avoid being associated with what is cis-heterodissident (Pereira, 2009; Bachmann and Gooch, 2018).

To address these personal consequences, a whole range of clinical intervention is available for non-heterosexual individuals through affirmative psychotherapy (APA, 2012). Moreover, to better understand the socio-cultural phenomenon and prevent it, there is an extensive tradition within social psychology for the study of attitudes (Eagly and Shelly, 1993). Attitude analyses related to internalized homonegativity shed more light on this.

One of the most commonly used instruments to measure sexist attitudes to date is the Ambivalent Sexism Inventory (ASI; Glick and Fiske, 1996). According to the theory underlying the ASI, sexism includes both hostile attitudes that directly accept that women are inferior and guileful and less explicit, benevolent attitudes that reinforce the view of femininity as delicate, defenseless, needy and available to men. These two dimensions act as ambivalences, drawing in women who personify traditional femininity and punishing them when they do not reproduce it, all from a position of “chivalry” (Glick and Fiske, 2011). At the same time, a contrary logic is argued in which women try to attract men in order to control them, while the women themselves are romantically objectified as indispensable to the completeness and happiness of the man. To reach this objective, the man presents himself as a provider and protector of the woman (Glick et al., 2004). Consequently, benevolent sexism in all its aspects may seem advantageous for heterosexual women (Hammond and Overall, 2013), who view the man who engages in it as attractive and desirable (Montañés et al., 2013).

Once this necessity is accepted as part of the measurement of sexism, it makes sense to consider the issues that the factor of heterosexuality may raise. As noted above, the influence of sexism on the rejection of anything that deviates from heteronormativity has been studied on several occasions. However, the connection between having a non-heterosexual orientation and sexist beliefs rarely appears in most of the research done (Blumell and Rodriguez, 2019; Cowie et al., 2019). In Spain, no studies on sexism have taken sexual orientation into account as a mediator variable. The studies that have adapted and validated the Ambivalent Sexism Inventory have not even considered the factor relevant to the content validity (López-Sáez et al., 2019).

Thus, the expression of levels of sexism among non-heterosexuals is underexplored. Although they are not as dependent upon heterosexuality, these individuals have suffered the consequences of sexism and may interiorize the attitudes and norms of the patriarchal society in which they grew up. While among heterosexuals, a low resistance to heterosexual norms indicates conservative standards regarding sexual morality and traditional gender roles (Badenes-Ribera et al., 2016), in homosexuals and bisexuals it may be related to a lack of acceptance of one’s own orientation as valid for happiness and/or completeness.

Several works have demonstrated the sexist attitudes of homosexual males. One of the defining aspects of hegemonic masculinity is the renunciation and rejection of the feminine (Anderson, 2009; Kimmel, 2017) and the avoidance of homosexuality (Anderson, 2009; Plant et al., 2014). Within anti-femininity, men are also categorized as having a homoerotic desire to be associated with female roles and/or positions (Lehavot and Lambert, 2007). In addition, the rejection of the feminine is a good predictor of anti-gay attitudes (Wilkinson, 2004; Parrott et al., 2011). Despite this, gay and bisexual men are not oblivious to the rejection of the feminine. According to Warriner et al. (2013), the integrated threat theory may explain how the transgression of the hypermasculinity established within the parameters of the sexism inherent in heterosexual desire is sanctioned through external and internal homophobia. In other words, among gays, the internalization of the construct of masculine femininity as a threat to maintaining social power generates a conflictive self-perception that holds the feminine in contempt and reinforces hegemonic masculinity (Gimeno, 2005; Rodriguez et al., 2016; Murgo et al., 2017; Lovelock, 2019). Thus, despite the evolution of masculinity toward less hegemonic positions, it seems that when men see their masculinity threatened, they react by returning to those more retrograde and hegemonic positions (Falomir-Pichastor et al., 2019).

As a consequence, the internalization of a sexist ethos within gays translates into negative attitudes, first toward women, through misogyny and the invisibilization of groups of women within the homosexual community (Trujillo, 2009; Martínez, 2017) and then toward themselves through internalized homophobia (Borrillo, 2001; Zheng et al., 2017). In fact, Moss (2002) draws a parallel between internalized homophobia and internalized misogyny in that both share a feeling of fear of the feminine. Additionally, these sexist beliefs translate into dynamics of intra-gender violence within homosexual couples as they reproduce heterosexual roles (Frost and Meyer, 2009; Barrientos et al., 2016; Li and Samp, 2019) and into the hierarchized sexual practices that determine who is considered superior (Zheng et al., 2017).

Other instruments also make it possible to measure the less commonly used construct of internalized homophobia and homonegativity. Most of them focus on the gay population (Mayfield, 2001; Currie et al., 2004) and, to a lesser extent, on lesbians (Szymanski and Chung, 2002) and bisexuals (Paul et al., 2014). Roughly speaking, the arguments differentiate between a more explicit or conscious homonegativity and a more implicit or subtle one. The former is based on beliefs that directly reject the non-heterosexual orientation, viewing it as inferior, perverted or abnormal (Mayfield, 2001). The latter demonstrates an *a priori* acceptance of sexual-emotional desire as long as it is homonormative, masculine and, to some extent, relegated to the private sphere (Ross and Rosser, 1996). Therefore, internalized homophobia is defined as “the result of consciously or unconsciously learning the homophobic prejudices, stereotypes, and behaviors that prevail in a heteronormative context” Morell-Mengual et al., 2016, p.1). One of the best-known tools is the Short Internalized Homonegativity Scale (Currie et al., 2004), whose Spanish adaptation and validation was done by Morell-Mengual et al. (2016). The tool is designed to measure subtle internalized negativity in homosexual men in three dimensions: public visibilization and identification as gay; comfort having gay emotional and social relationships; and sexual comfort based on rejecting myths.

As argued above, the presence of sexist attitudes among LGB individuals may be related to a non-acceptance of their own sexual identity, demonstrating a certain amount of internalized homonegativity. By the same token, this lack of self-acceptance, along with high scores for sexism – whether in the heterosexual or LGB population – seems to imply an adaptability to the standards of heterosexual normativity. Studying the dynamic between these constructs, therefore, involves considering the degree of acceptance and/or rejection of hegemonic sexual norms. In this respect, the Polymorphous Prejudice Scale (Badenes-Ribera et al., 2016) and, in particular, the Resistance to Heteronormative Expectations subscale can be a suitable tool to that end.

This *ex post* facto study (Montero and León, 2007) investigates the relationships between the construct of sexism and internalized homonegativity beyond the theoretical plane. It looks at the differences according to sexual orientation and gender identity and the importance of different variables such as resistance to heterosexual norms, political affiliation and contact with LGB individuals. Since this is an exploratory study, which seeks to intersectionally elucidate the construct of sexism among little-studied populations (such as LGB; López-Sáez et al., 2019), no hypotheses were made beforehand, following a proposal similar to that by (Blumell and Rodriguez, 2019).

## Method

### Participants

A total of 841 students participated from three Spanish universities: Complutense (UCM, *N* = 404), Autónoma de Madrid (UAM, *N* = 333) and Rey Juan Carlos (URJC, *N* = 104). The 1.6% of the participants who were trans, non-binary gender or identified as non-heterosexual and non-LGB were not considered in the data analysis due to the very small sample size. Therefore, the analyzed data correspond to 829 students.

In total, 78.2% of the participants were cis women and 20.7% cis men. The participant ages ranged from 17 to 60 (M = 20.78, SD = 4.02). 50% were in their first or second years of university and 50% in their third or fourth years.

### Instruments^1^

#### Sociodemographic Questionnaire

This included gender identity (1 = man, 2 = woman), sexual orientation (1 = heterosexual, 2 = LGB), age, academic year, lack of contact with LG and B individuals (1 = yes, 2 = no) and political affiliation (1 = left, 2 = center-left, 3 = center-right, and 4 = right-wing). With respect to the last of the four political groups, the proposal from Morrison and Morrison (2002) was followed, where the higher scores reflect greater conservatism. This single-element scale of political affiliation is reliable and valid (Gerbner et al., 1984).

#### Ambivalent Sexism Inventory (ASI)

We used the short version with the Spanish translation by Expósito et al. (1998). This 12-item instrument evaluates sexism through two subscales that measure hostile sexism (ASI-HS) and benevolent sexism (ASI-BS). Higher scores reflect more sexist attitudes. Rollero et al. (2014) reported a good overall alpha coefficient of 0.83. In this study, it is 0.82.

#### Short Internalized Homonegativity Scale (SIHS)

The short Spanish version contains 13 items designed to evaluate the internalized negativity of homosexuals and bisexuals. Its three subscales measure comfort with public visibilization and identification as gay (SIHS-PIH); comfort maintaining gay social and emotional relationships (SIHS-SOCC); and sexual comfort (SIHS-SEXC). Higher scores indicate a higher degree of negativity. Morell-Mengual et al. (2016) report an alpha coefficient of 0.80. In this study, it is 0.68.

Like other measurements, the SIHS was developed with a sample of gay men. Its Spanish adaptation included lesbian women, but never bisexual men and women. As such, the content of the articles assumes homosexuality and does not use inclusive and non-sexist language. For that reason, we modified the version to adapt it (for example, we changed “I feel comfortable when other people discover that I’m homosexual” to “I feel comfortable when other people discover that I am not heterosexual”).

#### Resistance to Heteronormative Expectations Subscale (PPS-RHE)

The Polymorphous Prejudice Subscale, which contains four items, was used. The aim of the PPS-RHE is to evaluate the degree of adherence to the conservative norms governing sexual morality and traditional gender roles. The items were adapted using inclusive language (for example, “I feel restricted by the social expectations that people have for my gender” was changed to “I feel limited by the social expectations that people have for my gender”). Higher scores reflect greater resistance to heteronormativity. Badenes-Ribera et al. (2016) report an alpha coefficient of .84. An identical alpha coefficient was obtained in this study.

#### Marlowe-Crowne Social Desirability Scale (MCSDS)

As a type of control, the short Spanish version was applied (Gutiérrez et al., 2015). This contains 18 items presented as assertions that are accepted or rejected using a true-false response format. Higher scores indicate greater social desirability. Gutiérrez et al. (2015) report an alpha coefficient of internal consistency of 0.76. In this study, it is 0.65.

### Procedure

The participants were selected using a stratified random sampling, with proportional allocation for each of the three universities. Out of a total population of 3,745 undergraduate psychology students, the sample size was determined for a confidence level of 95%, maximum variability and a maximum error of ±3%. The groups and participants from each academic year were selected at random. During the selection process, a proportional selection criterion was followed according to gender identities. At UCM and URJC, the selected individuals were contacted when attending face-to-face classes, while at UAM they were contacted by email. The rejection rate of the selected individuals was 30%. All the participants received the same instructions and were informed that their participation was voluntary and their responses confidential and anonymous. Before beginning, they had to read and accept the informed consent. Subsequently, they were provided with a link and went online to participate. The study was evaluated and reviewed by the UAM Research Ethics Committee, which gave its approval.

## Results

### Descriptive Statistics

71.9% self-identified as heterosexual, 23.3% as bisexual and 4.8% as homosexual. Due to the small number of homosexual individuals, they were grouped together with the bisexuals, leaving an LGB group that was 28.1% of the total. With regard to political affiliation, 42.2% identified with the left, 35.7% with the center-left, 19.5% with the center-right, and 2.5% with the right. Due to the low number of participants affiliated with the right and their ideological proximity to the center-right (Jurado and Riera, 2019), they were grouped with the center-right participants and reorganized as the “right spectrum,” with 21% of the total. Most said that there was a homosexual (90%) or bisexual (83.4%) in their family or friendship circles.

Firstly, descriptive statistics were calculated for the different variables. Then, the factor scores were calculated for each instrument using unrefined methods (in this case, the average of the items for each factor).

Table 1 shows the means and standard deviations by gender identity (man/woman), sexual orientation (heterosexual/LGB), and political affiliation (left, center-left, right-wing spectrum).

**TABLE 1:** Means and standard deviations by gender identity and sexual orientation.

**Left**	**Heterosexuals**				**LGB**			
	**Women**		**Men**		**Women**		**Men**	
	**M**	**DE**	**M**	**DE**	**M**	**DE**	**M**	**DE**
Hostile sexism (ASI-HS)	1.26	0.49	1.58	0.76	1.18	0.30	1.23	0.30
Benevolent sexism (ASI-BS)	1.75	0.61	2.08	0.98	1.67	0.49	1.89	0.65
Heteroresistance (PPS-RHE)	3.86	1.41	2.27	1.25	4.41	1.11	4.69	1.23
Public identification (SIHS-PIH)	–	–	–	–	2.47	0.89	2.61	1.07
Sexual comfort (SIHS-SEXC)	–	–	–	–	1.28	0.53	1.46	0.62
Social comfort (SIHS-SOOC)	–	–	–	–	1.76	0.55	1.94	0.88
Social desirability (MCSDS)	0.45	0.17	0.42	0.21	0.44	0.17	0.38	0.17
Centre-left								
Hostile sexism (ASI-HS)	1.55	0.56	2.13	0.84	1.40	0.59	1.62	0.69
Benevolent sexism (ASI-BS)	1.99	0.68	2.16	0.66	1.83	0.65	2.06	0.68
Heteroresistance (PPS-RHE)	3.20	1.41	2.74	1.34	4.22	1.29	4.82	1.58
Public identification (SIHS-PIH)	–	–	–	–	2.41	0.77	2.90	1.05
Sexual comfort (SIHS-SEXC)	–	–	–	–	1.29	0.48	1.95	1.10
Social comfort (SIHS-SOOC)	–	–	–	–	1.83	0.63	2.34	1.12
Social desirability (MCSDS)	0.41	0.15	0.44	0.18	0.46	0.17	0.43	0.18
Right-wing Spectrum								
Hostile sexism (ASI-HS)	1.85	0.72	2.77	1.05	1.99	0.94	1.92	0.97
Benevolent sexism (ASI-BS)	2.24	0.73	2.59	1.20	2.36	0.99	2.67	1.13
Heteroresistance (PPS-RHE)	2.56	1.48	2.32	1.36	4.15	1.91	4.88	0.95
Public identification (SIHS-PIH)	–	–	–	–	3.00	1.08	2.67	1.03
Sexual comfort (SIHS-SEXC)	–	–	–	–	1.70	0.70	1.92	0.52
Social comfort (SIHS-SOOC)	–	–	–	–	2.11	0.81	2.00	0.40
Social desirability (MCSDS)	0.44	0.16	0.45	0.19	0.39	0.17	0.56	0.00

### Intergroup Comparisons Using Generalized Linear Models

To study the differences in the different levels of the constructs measured, an analysis of covariance was carried out for each of the scales, using the following factors: gender identity (man and woman), sexual orientation (heterosexual and LGB), and political affiliation (left, center-left, right-wing spectrum). Social desirability was included as a co-variable in all the analyses. Due to the asymmetry of the dependent variables, the ANCOVAs were discarded for generalized linear models. The results according to each attitudinal variable are given below.

**FIGURE 1:**
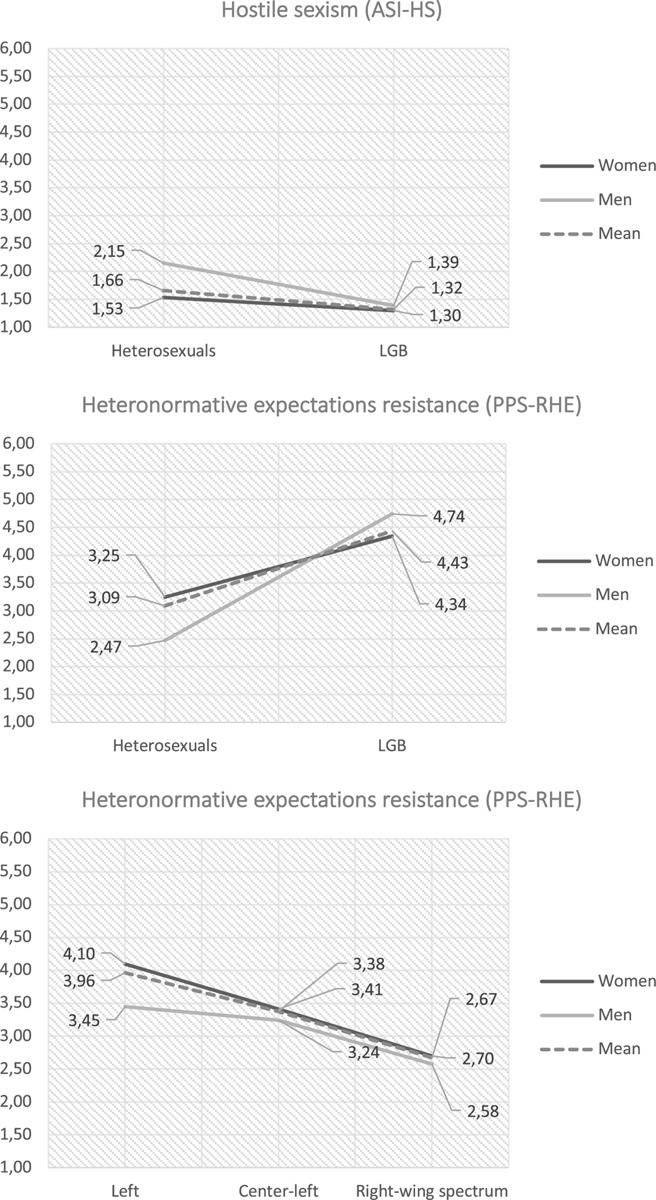
Graphs of significant interactions.

With regard to ASI-HS, the results show a significant effect in the interaction between gender identity and sexual orientation [*F*(1,816) = 12.45, *p* < 0.001, η*_*p*_^2^* = 0.02]. As seen in Figure 1 (upper section), this type of interaction requires a simple-effects analysis to be interpreted without error (see León and Montero, 2015). The simple-effects analyses for sexual orientation show that for both women [*F*(1,652) = 20.17, *p* < 0.001, η*_*p*_^2^* = 0.0]) and men [*F*(1,171) = 28.56, *p* < 0.001, η*_*p*_^2^* = 0.14], heterosexuals score higher than LGB for this type of sexism. On the other hand, the simple-effects analyses for gender identity show that for ASI-HS, there are only significant differences between heterosexual men and women [*F*(1,593) = 72.04, *p* < 0.001, η*_*p*_^2^* = 0.11], while the same does not occur between LGB men and women [*F*(1,230) = 1.21, *p* = 0.27, η*_*p*_^2^* = 0.01].

As political affiliation was not involved in any of the interactions, the primary effect was interpreted directly. In ASI-HS, then, people affiliated with the right-wing spectrum scored higher than those affiliated with the left [*F*(2,816) = 40.98, *p* < 0.001, η*_*p*_^2^* = 0.1].

There were no significant interactions in ASI-BS. As in ASI-HS, the primary effects of gender identity and political affiliation were significant in ASI-BS, with men scoring higher than women [*F*(1,816) = 9.28, *p* = 0.002, η*_*p*_^2^* = 0.01] and people on the right-wing spectrum scoring higher than those on the left [*F*(2,816) = 14.58, *p* < 0.001, η*_*p*_^2^* = 0.04].

Regarding PPS-RHE, the results show a significant effect in the interaction between gender identity and sexual orientation [*F*(1,816) = 15.19, *p* < 0.001, η*_*p*_^2^* = 0.02] and between gender identity and political affiliation [*F*(2,816) = 4.52, *p* < 0.05, η*_*p*_^2^* = 0.01]. As seen in Figure 1, the simple-effects analysis indicates that LB women have higher levels of PPS-RHE than heterosexual women [*F*(1,648) = 39.83, *p* < 0.001, η*_*p*_^2^* = 0.06] and GB men higher than heterosexual men [*F*(1,167) = 67.92, *p* < 0.001, η*_*p*_^2^* = 0.3]. In other words, regardless of gender identity, the level of PPS-RHE among LGB individuals rises in comparison with heterosexuals. On the other hand, regardless of their sexual orientation, tending toward the right produces lower levels of PPS-RHE in women [*F*(2,648) = 9.3, *p* < 0.001, η*_*p*_^2^* = 0.03]. There were no significant differences for men between the left and right [*F*(2,167) = 0.76, *p* = 0.5, η*_*p*_^2^* = 0.01]. The SIHS variables had no primary effects or significant interactions.

### Correlations and Multiple Regression Analysis

A bivariate correlation analysis was done, adding the “lack of contact” and “internalized homonegativity” (SIHS) variables with LGB. Correlations were estimated using Pearson’s coefficient and can be seen in Table 2, which shows the correlations of attitudinal and socio-demographic variables with ASI-HS and ASI-BS for LGB and heterosexual men and women.

**TABLE 2:** Correlations by gender identity and sexual orientation.

	**Heterosexual**				**LGB**			
	**Women**		**Men**		**Women**		**Men**	
	**Hostile sexism (ASI-HS)**	**Benevolent sexism (ASI-BS)**	**Hostile sexism (ASI-HS)**	**Benevolent sexism (ASI-BS)**	**Hostile sexism (ASI-HS)**	**Benevolent sexism (ASI-BS)**	**Hostile sexism (ASI-HS)**	**Benevolent sexism (ASI-BS)**
Heteroresistance (PPS-RHE)	−0.25**	−0.18**	–0.16	–0.16	–0.07	–0.07	0.07	0.09
Public identification (SIHS-PIH)	–	–	–	–	–0.03	0.07	0.22	0.41**
Sexual comfort (SIHS-SEXC)	–	–	–	–	0.27**	0.27**	0.36**	0.35*
Social comfort (SIHS-SOOC)	–	–	–	–	0.08	0.17*	0.14	0.41**
Political affiliation	0.36**	0.28**	0.47**	0.21*	0.40**	0.27**	0.43**	0.26
LG lack of contact	0.02	–0.01	0.18	0.14	–0.06	–0.04	0.04	–0.02
B lack of contact	0.19**	0.15**	0.28**	0.22*	–0.02	–0.03	0.18	0.19

*p < 0.05; **p < 0.01.

Additionally, a multiple regression analysis was carried out using the stepwise method. First, ASI-HS and ASI-BS were predicted based on gender identity, sexual orientation and political affiliation. Therefore, all the sociodemographic variables in the sample were used, except for the “contact” variables, which were discarded due to their unclear correlations.

Secondly, the predictions related to sexism in the heterosexual sample were made using PPS-RHE and political affiliation, while in the LGB sample, they were made using the SIHS (SIHS-PIH, SIHS-SEXC and SIHS-SOOC), PPS-RHE and political affiliation dimensions. For both samples, the analyses were done separating the LGB and heterosexual men and women.

The results of this analysis are shown in Tables 3–5.

**TABLE 3:** Multiple regression of sociodemographic variables.

	**Hostile sexism (ASI-HS)**			**Benevolent sexism (ASI-BS)**		
	**βa**	**R2**	**Change**	**βa**	**R2**	**Change**
Political affiliation	0.35	0.17a	0.17***	0.27	0.08a	0.08***
Gender identity	–0.44	0.24b	0.06***	–0.25	0.10b	0.02***
Sexual orientation	–0.17	0.25c	0.01**	–	–	–

*p < 0.05; **p < 0.01; ***p < 0.001.a: first predictor, b: second predictor adding the previous one, c: third predictor adding previous ones.

**TABLE 4:** Multiple regression as a function of variables in the heterosexual sample.

	**Hostile sexism (ASI-HS)**			**Benevolent sexism (ASI-BS)**		
	**βa**	**R2**	**Change**	**βa**	**R2**	**Change**
Women						
Political affiliation	0.31	0.13a	0.13***	0.24	0.08a	0.08***
Heteroresistance (PPS-RHE)	–0.15	0.15b	0.02**	–0.09	0.08b	0.01*
Men						
Political affiliation	0.47	0.22a	0.22***	0.21	0.03a	0.04*
Heteroresistance (PPS-RHE)	–0.17	0.25b	0.03*	–	–	–

*p < 0.05; **p < 0.01; ***p < 0.001.a: first predictor, b: second predictor adding the previous one, c: third predictor adding previous ones.

**TABLE 5:** Multiple regression as a function of variables in the LGB sample.

	**Hostile sexism (ASI-HS)**			**Benevolent sexism (ASI-BS)**		
	**βa**	***R*^2^**	**Change**	**βa**	***R*^2^**	**Change**
Women						
Political affiliation	0.26	0.14^a^	0.14***	0.22	0.11^b^	0.05**
Sexual comfort (SIHS-SEXC)	0.19	0.19^b^	0.05***	0.25	0.06^a^	0.07**
Men						
Political affiliation	0.23	0.15^a^	0.15**	–	–	–
Sexual comfort (SIHS-SEXC)	0.15	0.22^b^	0.07*	–	–	–
Social comfort (SIHS-SOOC)	–	–	–	0.32	0.17	0.17**

*p < 0.05;**p < 0.01;***p < 0.001.a:first predictor,b:second predictor adding the previous one,c:third predictor adding previous ones.

First, in the sample set, political affiliation, gender identity and sexual orientation have a good effect size, making it possible to explain 25% of the variance for ASI-HS. Something similar occurred with ASI-BS, although here sexual orientation lost its predictive value and the other variables only explained 10% of the variance.

Among heterosexual women, both PPS-RHE and political affiliation explained between 8% (for ASI-BS) and 15% (for ASI-HS) of the variance. Among LB women, political affiliation remained, but PPS-RHE lost predictive potential and SIHS-SEXC gained, with the variance percentage rising to between 11% (for ASI-BS) and 19% (for ASI-HS).

Among heterosexual men, PPS-RHE and political affiliation repeated prediction, with the variance increasing to 25% for ASI-HS, but only political affiliation had a low predictive potential for ASI-BS (3% of the variance). Among GB men, political affiliation and SIHS-SEXC explained 22% of the variance for ASI-HS, while 17% of the variance for ASI-BS was explained by SIHS-SOOC.

## Discussion

This study makes it possible to explore the differences between heterosexuals and LGB individuals with respect to the degree of sexism. It also facilitates a better understanding of other variables that may influence levels of sexism among both men and women in the heterosexual population and, specifically, the LGB population.

The results of the analysis of covariance reaffirmed the importance of gender identity (the fact of being a woman or a man) to differentiate the two sexisms. Additionally, these differences occur on the basis of sexual orientation (heterosexuals and LGB) and are more obvious in the sample of heterosexual men and women. Political affiliation is another variable that is a good indicator of the degree of sexism. In general, the results showed a lower degree of sexism among women, LGB individuals and people on the left.

These same groups, in turn, usually show greater resistance to adhesion to conservative norms regarding sexual morality and roles (PPS-RHE). This concurs with theories about the sex/gender/sexuality system (Westbrook and Schilt, 2014) that regard the heterosexual man as a privileged subject to the detriment of everyone else. According to this theory, a pyramid exists with different levels that enjoy a framework of privileges to a greater or lesser extent according to their proximity to the top, which is occupied by the cis man with a traditional, heterosexual masculinity.

For example, in our study, GB men with higher levels of ASI-BS than all the groups of women would be on a particular level of the pyramid. Moreover, the differences between groups regarding political affiliation (despite not being significant) indicate a slight trend. Even among people who belong to so-called “oppressed groups,” individuals on the right-wing spectrum have levels of sexism that are as high as those on the top of the pyramid. Therefore, it appears that despite reprisals for disturbing the *status quo*, LGB individuals can exhibit sexist attitudes when conservative parameters come into play.

These results show that being LGB and having sexist attitudes correlates with the components of internalized negativity, especially SIHS-SEXC. This means that the assumption of sexist canons can be associated with the belief that the correct sexuality adopts roles and morality in accordance with what is established by the heterosexual model (Warriner et al., 2013; Fogel, 2016). Moreover, at least in theoretical terms, there is a possibility that the components of sexism regarding heterosexual intimacy and gender differentiation stipulate a self-LGB-negativity. This sexism promotes non-identification and the negation of sexual and social interactions with LGB individuals.

The intersection of the gender identity variable influences the intensity of the correlations between SIHS and sexisms. Among GB men, for example, the correlations were higher and had greater significance. This could be related to the relationship that can be established between the consideration of femininity as a threat to the social role of GB men (ASI) and the fear of being associated with aspects of femininity linked to homoeroticism (SIHS). Here, individuals who participate in homonormative logic (López-Sáez, 2017) come into play, for example: “I am gay, but I am masculine and I like masculinity.” The fear of losing male privilege that is implied by femininity once again reveals the pyramid of social hierarchies (Taywaditep, 2002; Johnson and Samdahl, 2005).

In the heterosexual sample, both PPS-RHE and a lack of contact with bisexuals had the greatest correlations with sexism. With respect to PPS-RHE, this greater correlation and significance in the heterosexual sample could be due to the different implications related to breaking away from heteronorms. For heterosexuals, this split could entail a reconsideration of the traditional spheres of masculinity and femininity on which the components of sexism are based. LGB individuals, on the other hand, have no need to do this, and the low correlation seems to indicate a coexistence between resistance to heteronorms separate from sexist beliefs. However, among LGB individuals, this coexistence is associated with beliefs that produce self-stigmatization toward their own orientation in different areas and sexist beliefs.

With respect to the correlation between a lack of contact among heterosexuals with bisexuals and sexisms, it appears that contact could have an influence on protecting against sexism, at least among heterosexuals. However, the lower correlations regarding the lack of contact with LG individuals and the ambivalences in the correlations in the LGB sample suggest that this assertion be handled with a degree of caution. Contact with LGB individuals, who violate traditional gender expressions and roles, could be a protective factor by raising awareness about violence based on sexism. However, in societies like the Spanish one, where LGB individuals are widely accepted socially and easy to meet, simple contact is not always related to these protections (Badenes-Ribera et al., 2016; Lopes et al., 2017). Moreover, as Allport (1954) observes, contact with persons belonging to minority groups is not sufficient to reduce prejudice.

Again, the political affiliation variable is particularly important with respect to the correlation analyses. Political affiliation is significant in almost all the sample groups, both LGB and heterosexual women and men. This indicates the important influence of this variable. A large number of studies have identified the association between sexism and a right-leaning political affiliation (Warriner et al., 2013; Austin and Jackson, 2019). In this respect, predictive analyses make it possible to have a more comprehensive view of the degree of influence of this and other variables. The variable with the greatest predictive potential for both sexisms in the sample set was political affiliation, followed by gender identity. Sexual orientation added only very slightly to the variance level and only in the case of ASI-HS. In the heterosexual sample, PPS-RHE also added a very small variance, which was null for ASI-BS and among men.

In the LGB sample, SIHS-SEXC was the variable that most often improved the variance level explained by political affiliation. However, this was not the case for the group of GB men, since only SIHS-SOOC was an adequate predictor for ASI-BS. The lack of prediction for ASI-BS by political affiliation among GB men could be important, since it would indicate that a change in political affiliation in this group and for this sexism is not as relevant as SIHS-SOOC.

## Conclusion

A whole host of works have shown how sexism permeates different gender identities, cultures, studies and professions. There is an essential need to include an intersectional perspective in empirical research in order to obtain a comprehensive and holistic view of sexism. Intersectionality facilitates analyses that visibilize the different mediating and predictive variables that come into conflict or form part of the “bricks of the wall of oppression” (Ahmed, 2018). In this context, this study sheds light on the state of the question and the limitations.

First, the clear shortage of works that explore the question of sexual orientation with a focus on analyzing sexism in the LGB population was the motivating factor behind this study. Here, the analyses have revealed – contrary to reasonable logic – how a group that is subjected to oppression itself can at the same time hold negative attitudes toward other groups and even toward itself. In other words, it appears that LGB individuals learn negative hegemonic attitudes about women and/or femininity in a way that is similar to the interiorization of negativity and they support them, regardless of the harmful impact on their own wellbeing.

Additionally, the study of sexism in LGB individuals has raised some thought-provoking questions. The ASI to measure ASI-BS considers the dimension of heterosexual intimacy for a population that does not practice that intimacy. While it is true that LGB individuals could adopt sexist internalization for themselves and view this intimacy as suitable, they might not, instead accepting other ASI-BS parameters such as the care and protection of women and the feminine. However, in the latter case, although homosexual men have some levels of ASI-BS, they will always score lower than heterosexual men. This is a point of interest in terms of homonormativity, in which resistance to heterosexuality is assumed, but the other components of sexism are accepted and, consequently, the feminine is disparaged.

Secondly, the results indicate that being a psychology student is no guarantee of being free of sexist attitudes, of recognizing the pressures of dominant sexual normativity, or of having less self-stigma (in the case of being LGB). Although the current situation regarding the skills and information gap surrounding the construct of sexism has been brought to light here, no meta-analysis was done with other works that would have produced a detailed comparison of its extent.

All of these discoveries and questions require further study to better understand the complex relationships and mechanisms related to sexism and internalized LGB-negativity, particularly in the LGB community. Resolving the limitations related to the random selection in our study (without being able to consider sexual orientation beforehand – 596 heterosexuals, 40 homosexuals and 193 bisexuals) and increasing the sample size are necessary steps for future work. An exploration of more heterogeneous probability samples would also be beneficial, given that the results are only representative of the public university system in the Community of Madrid. In that respect, the population of psychology students is feminized, does not adhere to very conservative ideologies and is far from ethnically and culturally diverse.

Additionally, future directions should review the ASI to assess an adaptation of the scale for people without heterosexual desire. Similarly, improving the use of the variables by incorporating new ones or reconfiguring the existing variables (by evaluating the type of personal contact connection, for example) is called for. Finally, longitudinal studies would make it possible to contrast how learning evolves in psychology and to evaluate the changes that may occur as the student progresses through the program.

In conclusion, the benefits of exploring a broader panorama of the factors that intersect in sexism would lead to better diagnoses and a more holistic understanding of the phenomenon.

## Data Availability Statement

The raw data supporting the conclusions of this article will be made available by the authors, without undue reservation.

## Ethics Statement

The studies involving human participants were reviewed and approved by Comité de Ética de la Investigación de la Universidad Autónoma de Madrid. The patients/participants provided their written informed consent to participate in this study.

## Author Contributions

ML-S was the principal author and the one who has contributed most to the manuscript presented here, was elaborated the theoretical framework that supports the article, as well as the realization of the different analyses that were presented. DG-D was one of the contributors who have focused on making contributions to the theoretical framework and the final discussions. IM was one of the contributors who focused on making contributions to the statistical analysis and discussions derived from it. All authors contributed to the article and approved the submitted version.

## Conflict of Interest

The authors declare that the research was conducted in the absence of any commercial or financial relationships that could be construed as a potential conflict of interest.

## Footnotes

1 All of the scales except for the sociodemographic questionnaire and the Social Desirability Scale employed a 6-point Likert scale, ranging from 1 (strongly disagree) to 6 (strongly agree), in order to avoid neutral answer trends and to homogenize the survey information.
